# Memory as Medicine^1^

**DOI:** 10.3201/eid1802.AC1802

**Published:** 2012-02

**Authors:** Polyxeni Potter

**Affiliations:** Centers for Disease Control and Prevention, Atlanta, Georgia, USA

**Keywords:** art science connection, emerging infectious diseases, art and medicine, Radcliffe Bailey, En Route, memory as medicine, cholera, influenza, mixed media, slavery, American art, about the cover

**Figure:**
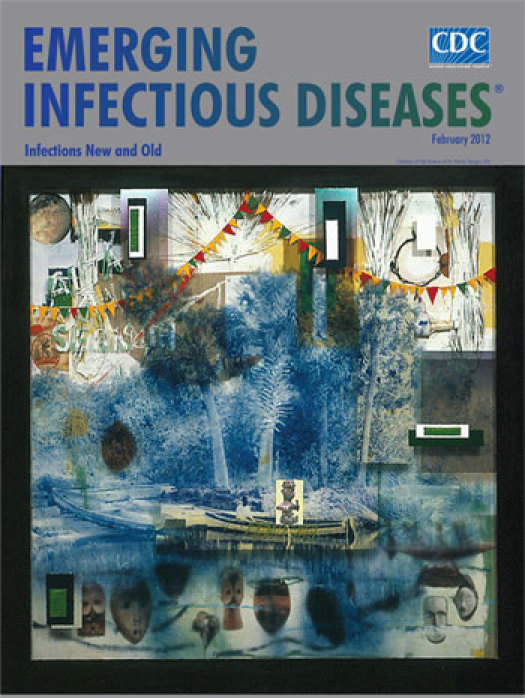
**Radcliffe Bailey (b. 1968) *En Route* (2005) Photograph on Plexiglas, coconut palms, felt, acrylic, and wood (172.7 cm × 172.7 cm × 14 cm)** Collection of High Museum of Art, Atlanta, Georgia, USA

“The true art teacher I had was my mother,” Radcliffe Bailey admits, “She led me to the well.” With the family’s encouragement, the artist started his creative journey early in life, “doodling and drawing,” at first in New Jersey, where he was born, and after the family moved to Georgia, in the Atlanta College of Art, next door to the High Museum of Art, where he ran into the likes of Jacob Lawrence and Romare Bearden. “I’ve learned a lot from this museum, learned a lot about all different types of art.” And “When you have the opportunity to see an artist like Jacob Lawrence… you can foresee it. You have the opportunity to think… this could actually happen [to me].”

Family and community remain a powerful force in Bailey’s life and work. Layers of childhood memories blend with historical events to form the rich imagery of his large mixed media paintings and installations. These works are filled with tintypes of relatives, everyday objects, fanciful figurines, and Georgia red clay. “I use Georgia clay because it’s in my backyard. My backyard is on Civil War grounds―boom. Then I trace that to family members that were in the Civil War… and to my father being a railroad engineer.” “Atlanta has this interesting past that makes you want to dig deeper and understand what was once there, even though it may be covered…. Sherman burnt down the city. They say when you want to get rid of something, you burn it, but you don’t really get rid of it. I can look out my back door and see a lot.” As for style, “For me, it’s always been about having conversations with everyone. I don’t want to make work that’s above, that speaks a certain way that a common person couldn’t understand. I’m more concerned with having that conversation.”

“I always come to painting as a sculptor,” says Bailey, whose early art training was in sculpture and inspiration from such modern practitioners as Martin Puryear. “Everything is based on materials.” “Painting came to me at the last minute…. That’s why my paintings seem constructed. I am interested in questioning. What is painting? What is sculpture? I can build up paint so it feels like sculpture.”

The language of music is of interest. “I’ve been listening to the sound of the wind and I’ve been listening to my dogs bark at night on a full moon. Of course there are certain jazz musicians and female singers I listen to.” “I love Sun Ra’s music…. [He] brings together a mixture of time periods, and he fuses them together into something that sounds futuristic. I like to compare my work to that in terms of the sounds and the riffs of different times.” And old objects are of great interest. He collects these found objects and adapts them to his work. “It’s very much like hip-hop, patching and putting things together quiltlike, using old things to make new things.”

“I’ve always felt like the only way I can heal myself throughout certain things is to go back through my memory, learn from memory.” This travel through memory seems pertinent to *En Route,* on this month’s cover. This work is one in a cluster with a historical theme: slavery’s Middle Passage or the middle leg in the journey from Africa to a final destination, during which for 50 days or more, slaves were caged in suffocating quarters under inhumane conditions on the way to the West Indies, North America, South America, and Europe. The work contains several symbolic elements shared in the cluster: Water; the boat; blue color; tropical imagery; navigational tools (the sun, the moon, the planets).

“I started making these pieces that were very boxlike. When people look at them they think, ‘Oh, those are some real big frames,’ but they’re actually constructed as medicine cabinets. The idea was… you go into your medicine cabinet to find something to heal you. And I always felt like my memory was my medicine.”

With its Plexiglas surface and thick framelike perimeter, *En Route* has the feel of peering through a window into a luscious tropical scene made in eerie cobalt blue. The glasslike transparency of the water surface and the haunting array of African masks suspended in it add to the dreamlike unreality. Yet behind the water pool is a real figure in a boat ready to depart. And above the vegetation on the horizon, Bailey scatters clues―travel by sea, a musician, whose work transcends the sadness of historical fact; strings of dressing lines alluding to Marcus Garvey’s 1920s Black Star Line, an enterprise intended to provide a better way to travel and trade around the Atlantic; letters; and architectural blocks with blank openings, perhaps suggesting “the door of no return” on Gorée Island in Senegal. “Think about a dream. How could I articulate a dream and make it make sense? I think the dream has so many places where you can enter and break away. My work is like a never-ending dream. And it is very cryptic.”

“My desire is just to make art, to make things,” Bailey says when asked to explain his work. “I like to think of my artworks as similar to the music of Thelonius Monk…. They're like rituals that just happen.” Yet, just as you can scarcely separate the author of a novel from the story or the scientist from the experiment, you can hardly appreciate art apart from its source. The painter’s life and times, knowledge of the world and outlook on life are an integral part of the artwork. And in Bailey’s case, the choice of artifacts and the connections made between them create not an archive but a progression of history, which is why a work that recounts a sad chapter can actually be uplifting.

Layers of cryptic historical data feature too in science and are as essential to appreciating it as those of any painting in any era. Just as the specter of slavery, which has been exhaustively examined, could never be entirely resolved without continued historical scrutiny, emerging disease problems would be far more difficult to decipher outside their past lives and times. Not much has changed, for example, in the medicine cabinet as we look for ways to heal cholera. Lack of potable water and sanitation remain the principal reasons why cholera exists today and largely in Africa.

But much has been learned about pandemic influenza from examining the 1918 pandemic. And not just by revisiting it. Like the artist, public health practitioners are constantly reevaluating the pandemic, not quite knowing where new outlooks might take them. They resemble Radcliffe Bailey. “I'm like free jazz. I'm not concerned about where I'm going, just as long as I'm moving forward and documenting life.” We benefit from reviewing old scourges: slavery, cholera, influenza. Each time we revisit them is like revisiting the art museum to see the old masters. We see something new. Not because the old masters have changed so much but because with new knowledge and outlook, we have.

## References

[1] Thompson C, Barilleaux RP, Diawara M, Rooks M, Spriggs SE. Radcliffe Bailey: memory as medicine exhibition catalogue. Seattle (WA): Marquand Books; 2011.

[2] Caldwell K. Radcliffe Bailey [cited 2011 Dec 8]. http://paulsonbottpress.com/about/oktp/oktp_bailey.pdf

[3] Radcliffe Bailey [cited 2011 Dec 8]. http://www.high.org/Art/Exhibitions/Radcliffe-Bailey.aspx

[4] Shanks GD, Brundage JF. Pathogenic responses among young adults during the 1918 influenza pandemic. Emerg Infect Dis. 2012;18:201–7. 10.3201/eid1802.102042 22306191 PMC3310443

[5] Morens DM, Taubenberger JK. 1918 Influenza, a puzzle with missing pieces. Emerg Infect Dis. 2012;18:332–5. 10.3201/eid1802.111409 22304897 PMC3310470

[6] Echenberg M. Africa in the time of cholera: a history of pandemics from 1817 to the present. New York: Cambridge University Press; 2011.

